# Protocol for the fixation of nitrogen from air using focused ultrasound

**DOI:** 10.1016/j.xpro.2026.104499

**Published:** 2026-04-20

**Authors:** Lukman A. Yusuf, Shaun Fletcher, Paul Prentice, Mark D. Symes

**Affiliations:** 1 School of Chemistry, University of Glasgow, G12 8QQ Glasgow, UK; 2 James Watt School of Engineering, University of Glasgow, G12 8QQ Glasgow, UK

**Keywords:** Physics, Energy, Chemistry

## Abstract

Nitrogenous fertilizers, containing nitrate as a key component, are important soil-enriching compounds that support crop growth and yield. Here, we present a protocol for the ultrasonic fixation of nitrogen from air, using focused ultrasound in water. We describe steps for designing, assembling, and configuring the sonoreactor. We detail procedures to achieve the optimal nitrate concentration and pulse width for the applied ultrasound and to apply these conditions to a benchtop reactor to form micromolar concentrations of both nitrite and nitrate.

For complete details on the use and execution of this protocol, please refer to Yusuf et al.^1^

## Highlights

• Steps for using pulsed focused ultrasound to induce cavitation for nitrogen fixation
• Procedures for quantifying nitrogen fixation products (nitrite and nitrate)
• Description of the optimization of the experimental setup and parameters
• Steps for applying optimized results to a bench-scale prototype

## Abstract (editor highlights)

Publisher’s note: Undertaking any experimental protocol requires adherence to local institutional guidelines for laboratory safety and ethics.

## Graphical abstract

**Figure:**
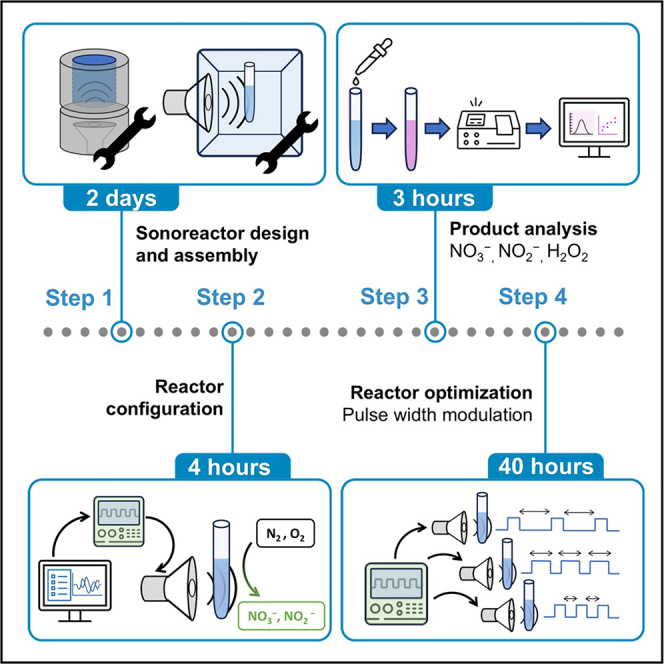

## Before you begin

Nitrogenous fertilizers are a key class of agricultural inputs that supply plants with nitrogen, which is an essential nutrient for plant growth and crop yield. Nitrogen supports vital functions such as chlorophyll formation, protein synthesis, and overall vegetative development. Commonly-applied forms of nitrogenous fertilizers include nitrate (NO_3_^-^), ammonium (NH_4_^+^), and urea.^2^

The fixation of atmospheric nitrogen (N_2_) into useful compounds like ammonia (NH_3_) and nitric acid (HNO_3_) is a highly challenging process due to the strong triple bond in N_2_, which makes it extremely inert.^3^ Traditionally, the Haber-Bosch process has dominated industrial nitrogen fixation, converting N_2_ to ammonia using hydrogen at high temperatures (350–500°C) and pressures (around 200 atm).^4^ While effective at large scales, this method requires costly infrastructure and depends on hydrogen supply. Furthermore, the production of nitric acid is generally performed via the Ostwald process, which re-oxidizes ammonia at very high temperature (600–900°C).^5^ Ultrasound-driven nitrogen fixation is an alternative, environmentally friendly approach that can be used to fix nitrogen from air. Our recent manuscript (Yusuf et al., 2025) studied this process from both a fundamental and an applied perspective. In the protocol below, we describe how nitrogen can be optimally fixed using ultrasound delivered by pulse-width modulation transducer excitation. We detail the analytical techniques used to benchmark the processes in terms of nitrate and nitrite production. Additionally, to demonstrate the wider applicability of this technique, we also specify the design of an inexpensive sonoreactor which can convert nitrogen from the air into nitrate and nitrite on the benchtop scale.

Before beginning any nitrogen fixation experiments, ensure that the analytical reagents are prepared (as outlined in materials and equipment), the reactor is correctly wired and assembled, and that there is sufficient degassed ultrapure water available. Descriptions of these steps are outlined below.

### Innovation

The key innovation in this research is the optimal utilization of acoustically generated cavitation for nitrogen fixation at an efficacy not previously achieved. This is made possible using a focused ultrasound transducer, which concentrates the acoustic cavitation within the sample volume. Additionally, the use of pulsed ultrasound to induce cavitation has been shown to significantly enhance the yield of nitrate and nitrite compared to continuous operation. The application of both focused and pulsed ultrasound has received limited attention in previous ultrasonic nitrogen fixation studies. In combination with high-speed imaging, the configuration reported herein enables precise control and optimization of the cavitation process and facilitates accurate implementation of the excitation protocol. Furthermore, the findings from the optimization procedure are successfully translated herein into a novel prototype device, which is capable of fixing nitrogen based on the excitation parameters identified through the optimization procedures.

### Preparing degassed ultrapure water

**Timing: 24 h**

1. Prepare degassed water for filling the sonoreactor.
  a. Heat 12 L of ultrapure water to just below 100°C.
    ***Note:*** Ultrapure water typically has a resistivity of 18.2 MΩ cm at 25°C, with less than 5 ppb of total organic carbon. If a lab water purification system is unavailable, other ultrapure water sources (e.g. HPLC, LC-MS grade water) can be used.
  b. Transfer the liquid immediately to each of the 10 (1 L media) bottles with a screw-top lid. Fill to the brim and seal.
  c. Store the sealed bottles for 24 h.

### Preparing the focused ultrasound reactor

**Timing: 2 days**

**Figure 2:**
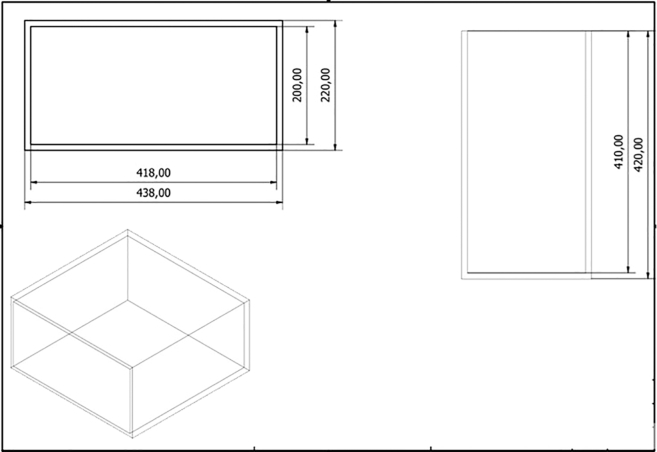
The engineering drawing for the sonoptic tank It was constructed using acrylic of thickness 10 mm. The dimensions in the figure are given in mm.
  b. Position the focused ultrasound transducer in the sonoptic tank in such a way that the focal region lies in the center of the tank.
    ***Note:*** To locate the focal region, scan the acoustic field using a 0.2 mm calibrated needle hydrophone. Scan at 225 kHz, 10 mV_pp_, 50 cycles, with a scan grid spacing of 0.5 mm and a measurement depth of 30 mm. Map the volume in front of the transducer and identify the region with a high pressure profile. This region is the focus of the transducer. The focal length should match the values specified by the manufacturer of the transducer. An example of a pressure field map is shown in Figure 3.
  c. Fabricate a quartz glass tubular “sonoreactor” with a sintered disk at the base.
    ***Note:*** A tube with a diameter of 10 mm, a length of 130 mm, thickness of 1 mm, and a sintered disk of porosity 3 was used in the reactor shown in Figure 1.
  d. Connect a compressed air cylinder via rubber tubbing to the bottom of the sonoreactor so that air can pass through the sintered disk into the main chamber.
  e. Position the sonoreactor vertically at the focal region of the transducer.
  f. Set a high-speed camera perpendicular to the transducer to capture cavitation within the focal region.
    ***Note:*** For capturing cavitation, ensure that the camera can operate at *20,000* frames per s (fps), with a 159 ns shutter time. The Supplemental Information of the associated primary research manuscript (Videos S3, S4 and S5) shows examples of such images.^1^
  g. Place a shockwave passive cavitation detector (swPCD) immediately behind the sonoreactor relative to the transducer.
    ***Note:*** This measures the bubble collapse shockwave emissions from cavitation with some interference from the driving acoustic field. The swPCD used in the associated primary research manuscript was custom-made. The development and characterization of this swPCD have been published.^6^ Commercially available passive cavitation detectors may be used as an alternative but should be suitably characterized prior to use.
  h. Fill the sonoptic tank with degassed water.

2. Assemble the focused ultrasound reactor for optimization experiments, as shown in Figure 1.
  a. Fabricate a “sonoptic tank” from polypropylene or acrylic.
    ***Note:*** A tank measuring 420 × 438 × 220 mm made from 10 mm acrylic was used in the manuscript this protocol is based on.^1^ However, the exact size of the sonoptic tank is not important provided that both the transducer and sonoreactor can fit. See Figure 2 for an engineering drawing of the sonoptic tank.

**Figure 1:**
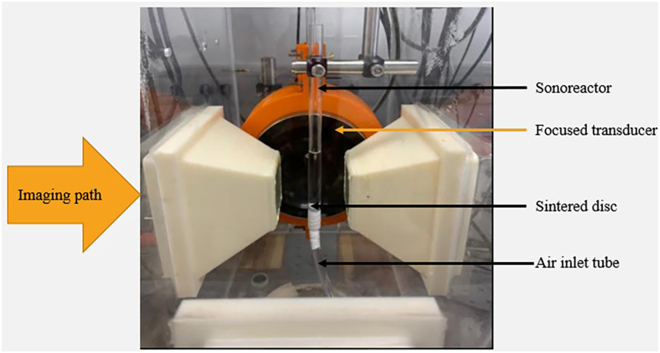
The ultrasound reactor for optimization experiments, showing the relative positioning of the transducer, sonoreactor, and imaging path within the sonoptic tank
3. Connect the transducer to the waveform generator and power source, according to Figure 4.
  a. Connect the waveform generator via Channel 1 to a power amplifier.
  b. Link the outwards connection of the amplifier to the focused ultrasound transducer via an impedance matching network.
  c. Link the sync-out of the waveform generator to the delay generator.
  d. Attach both the high-speed camera and the passive cavitation detector (via an oscilloscope) to the delay generator across two trigger relays.

**Figure 3:**
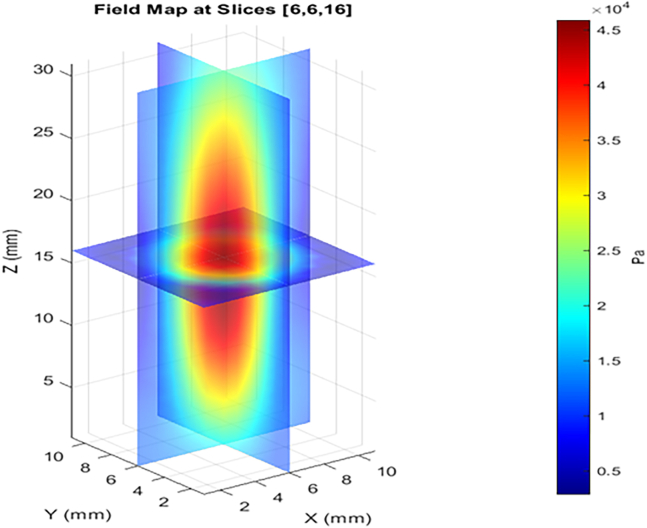
A typical pressure field map for the focused transducer (H-149) 3-D scanned at 225 kHz, 10 mVpp, 50 cycles The scan grid spacing used is 0.5 mm with a measurement depth of 30 mm.
4. Program the waveform generator, delay generator, and oscilloscope.
  a. In Channel 1 of the waveform generator (connected to the transducer):
    i. Set the waveform to a sine wave.
    ii. Set the frequency to 200 kHz.
    iii. Set the amplitude to 1000 mVpp (V_pp_ = peak-to-peak voltage).
    iv. Set “Burst” to the desired pulse duration (in terms of the number of cycles).
    v. Set trigger to “External”.
  b. In Channel 2 of the waveform generator (connected to the external trigger):
    i. Set the waveform to “pulse”.
    ii. Set the period to the pulse length plus the desired pulse interval.
    iii. Set the amplitude to 5 V_pp_ with an offset of 2.5 V.
    iv. Set the “leading” to 0.
    v. Set the burst to the desired pulse duration (in terms of the number of cycles).
    vi. Set trigger to “Manual”.
      ***Note:*** Make sure both output channels are off when programming the waveform generator.
  c. Set the delay generator so that both output channels (triggering the camera and oscilloscope respectively) are set to activate simultaneously to initiate data collection.
  d. Set the oscilloscope to collect acoustic emissions data with the swPCD at a sampling rate of 25 mega samples per second.

### Preparing the bespoke bench-scale sonoreactor

5. Design and fabricate a sonoreactor from stainless steel. A representative example used in the primary research for this protocol is described below.
  a. Ensure the housing for the reactor is of the appropriate size for the transducer.
    ***Note:*** The 40 kHz transducer used in this protocol measures 54 mm (height) × 45 mm (base diameter) × 35 mm (diameter of top face).
  b. Ensure the reactor chamber holds an appropriate volume of water.
    ***Note:*** The volume of the prototype reactor shown in Figure 5 is 100 mL.

**Figure 4:**
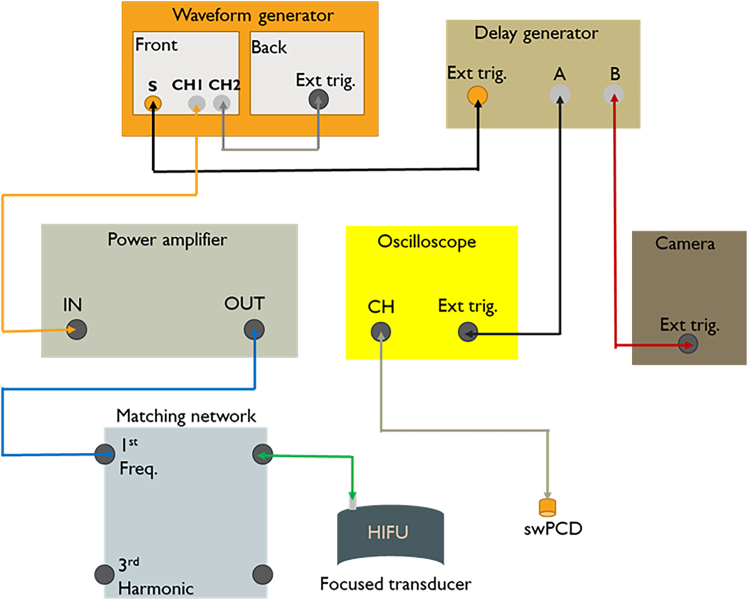
Electrical connections between the components of the focused ultrasound reactor

**Figure 5:**
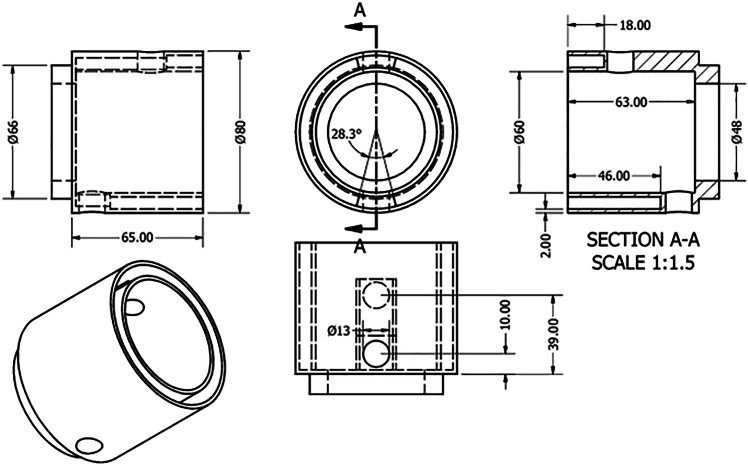
Schematics of the prototype sonoreactor used in the primary research paper associated with this protocol (including the lid and cooling jacket mechanism) All measurements are in millimeters.
  c. Add a cooling mechanism to prevent overheating of the components.
    ***Note:*** The example below is a jacketed reactor, which allows for cold water to flow around the reactor chamber.
  d. Design the reactor such that it can be made airtight with the addition of a lid and seal.
    **CRITICAL:** The lid should have two ports through which air can be introduced into, and vented from, the reactor chamber. This allows for experiments with both continuously aerated and degassed water.
  e. Connect the transducer to the outside of the base of the reactor chamber with a screw and seal in place with epoxy resin.
  f. Allow the epoxy glue to be cured for 24 h.
    **Pause point:** After 4–6 h, the epoxy glue will have set, and the device can be handled. The device should not be tested until the epoxy has fully cured.
6. Connect the transducer to a piezo driver to power the sonoreactor.
  ***Note:*** For a 40 kHz, 50 W transducer, a 150 W piezo driver is appropriate (PDUS210 Ultrasonic Driver).
  a. Connect the transformer to the transducer.
    i. Select the transformer based on the impedance of the bespoke reactor.
      ***Note:*** A transformer with turn ratio 13.6 was used based on the impedance (231 Ω) and Q factor (54.1) of the benchtop sonoreactor used in the associated primary research paper. The Supplemental Information of the primary research paper (Figure S9) shows a typical impedance spectrum.^1^
    ii. Connect the two wires from piezo driver to the transformer, and the output of the transformer to the electrodes of the transducer.
  b. Use “sweep mode” to probe a range of frequencies around the resonance frequency of the reactor.
    i. Monitor the impedance graph of the transducer to ensure that the resonance frequency is as expected (i.e., 40 kHz).
    ii. Note the phase.
  c. Program the piezo driver for pulse mode.
  d. Activate “phase tracking” in the control software.
  e. Set the maximum power, voltage, and current.
  f. Set the transformer turns to match the transformer used.
  g. Set the voltage to the desired value (i.e., 250 mV_pp_).
  h. Set the frequency to the value from the previously obtained impedance graph. Set the minimum and maximum values to approximately 100 Hz either side of this value.
  i. Set the phase to the value obtained from the frequency sweep.
  j. Power-on the reactor and make sure that the key parameters (output voltage, output current, amplifier power, load power) remain steady.

## Key resources table

**Table:** 

REAGENT or RESOURCE	SOURCE	IDENTIFIER
**Chemicals, peptides, and recombinant proteins**		

Air, Industrial Grade	BOC	12-W
Sulfanilamide, 98%	Alfa Aesar	CAS#63-74-1
N-(1-Naphthyl)ethylenediamine dihydrochloride, 96%	Alfa Aesar	CAS#1465-25-4
Sodium nitrite, 99.999% (metals basis)	Thermo Fisher Scientific	CAS# 7632-00-0
Potassium nitrate, 99.994%	Thermo scientific	CAS#7757-79-1
Potassium iodide, ≥99.0%	Sigma-Aldrich	CAS#7681-11-0
Iodine, ≥99.99% trace metals basis	Sigma-Aldrich	CAS#7553-56-2
Ammonium heptamolybdate tetrahydrate, 99%	Alfa Aesar	CAS#12054-85-2
Hydrogen peroxide solution (30 wt.%)	Sigma-Aldrich	CAS#7722-84-1
Hydrochloric acid, 37%, analytical grade	Fisher Scientific	CAS#7647-01-0

**Software and algorithms**		

Origin 2025	OriginLab	https://www.originlab.com
MATLAB	MathWorks	https://www.mathworks.com/products/matlab.html

**Other**		

Ultrasound transducer	Allendale Ultrasonics	40 kHz, 50 W
Piezo Driver	210 W	pdus210
High intensity focused ultrasound (HIFU) transducer	Sonics	https://sonicconcepts.com/therapy-transducers/
Oscilloscope	Tektronix	https://www.tek.com/en/products/oscilloscopes/5-series-mso
Digital delay generator	DG535	https://www.thinksrs.com/products/dg535.html
Signal generator	Rigol	https://www.rigol-uk.co.uk/product/rigol-dg4102-100mhz-function-arbitrary-waveform-generator/
RF power amplifier	E & I	https://rfamplifiers.eandiltd.com/item/rf-amplifiers/rf-amplifiers-1/1040l
Needle hydrophone	Precision Acoustics	NH0200
High speed camera	Photron	FASTCAM SA-Z 2100 K (128 GB RAM)
Passive cavitation detector	Sonic Concepts	Y-107
UV-vis spectrophotometer	Agilent	Agilent Cary 60
Total dissolved gas sensor	OTT Hydromet	https://www.ott.com/products/sensors-108/total-dissolved-gas-tdg-sensor-163/
Epoxy glue	J-B Weld	https://www.jbweld.co.uk/

## Materials and equipment

**Table:** 6 M hydrochloric acid solution

Reagent	Amount
Hydrochloric acid (37%)	123.2 mL
Ultrapure water	126.8 mL
**Total**	**250 mL**

Store in a well-ventilated area away from direct sunlight.

**Table:** 1 M hydrochloric acid solution

Reagent	Amount
6 M hydrochloric acid solution	16.7 mL
Ultrapure water	83.3 mL
**Total**	**100 mL**

Store in a well-ventilated area away from direct sunlight.

**CRITICAL:** Hydrochloric acid is highly corrosive, and can cause severe skin burns and eye damage. All preparations involving concentrated hydrochloric acid should take place in a contained environment (e.g. a fume cupboard), and the hydrochloric acid should be stored in a well-ventilated area away from heat and light. Personal protective equipment, including gloves, safety glasses, and a lab coat, should be employed.

**Table:** Griess coupling agent (0.1 wt% N-(1-naphthyl) ethylenediamine) solution

Reagent	Amount
N-(1-naphthyl) ethylenediamine	0.25 g
Ultrapure water	250 mL
**Total**	**250 mL**

Store at <4°C in an amber glass container for up to one month.

**Table:** Griess sulfanilamide (0.2 wt% 4-aminobenzenesulfonamide) solution

Reagent	Amount
Sulfanilamide	0.5 g
Ultrapure water	250 mL
**Total**	**250 mL**

Store at <4°C for up to two months.

**Table:** 0.1 M nitrite solution

Reagent	Amount
Sodium nitrite	0.69 g
Ultrapure water	100 mL
**Total**	**100 mL**

**Table:** 0.1 M nitrate solution

Reagent	Amount
Potassium nitrate	1.01 g
Ultrapure water	100 mL
**Total**	**100 mL**

**Table:** 0.1 M potassium iodide solution

Reagent	Amount
Potassium iodide	4.15 g
Ultrapure water	250 mL
**Total**	**250 mL**

Store in amber glass.

**Table:** 10 mM triiodide solution

Reagent	Amount
Iodine	0.64 g
0.1 M potassium iodide solution	250 mL
**Total**	**250 mL**

Store in amber glass.

**Table:** 0.01 M ammonium heptamolybdate solution

Reagent	Amount
Ammonium heptamolybdate tetrahydrate	0.62 g
Ultrapure water	50 mL
**Total**	**50 mL**

Store in amber glass.

**Table:** 0.1 M hydrogen peroxide solution

Reagent	Amount
30 vol% hydrogen peroxide solution	0.255 mL
Ultrapure water	24.745 mL
**Total**	**25 mL**

Store at <4°C in amber glass.

## Step-by-step method details

### Quantification of nitrite ions in water

**Timing: 1 h**

This step describes the standard technique of nitrite ion quantification in aqueous samples. The specific procedure for the test, as well as instructions for creating a calibration curve, are presented. The limit of detection for the procedure described below is 0.1 μM.

1. Perform the Griess test for nitrite ions.
  a. Add 5 mL of Griess sulfanilamide solution to a 20 mL glass vial.
  b. Add 1 mL of 6 M HCl to the vial and agitate for 1 min.
  c. Add 5 mL of the sample to be tested.
  d. After 2 min, add 1 mL of the Griess coupling agent solution to the vial.
  e. Leave the vial to stand for 20 min.
  f. Collect a UV-vis spectrum of the sample between 600 nm and 500 nm and note the absorbance of the peak at ʎ_max_ = 540 nm.

**CRITICAL:** Ensure that a baseline measurement of the ultrapure water used in the sonication experiments is taken before analyzing any samples.

**Figure 6:**
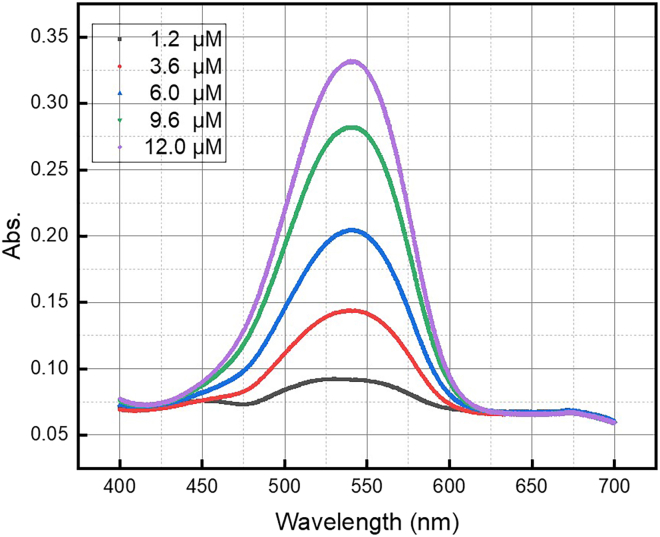
Example UV-vis spectra from the Griess test calibration process for nitrite quantification The absorption at ʎ_max_ = 540 nm should be used for the calibration curve.

2. Prepare a calibration curve with solutions of known nitrite concentration.
  a. Starting from a 0.1 M nitrite solution, perform three sequential 10-fold dilutions to obtain 50 mL of 100 μM nitrite solution.
  b. From the 100 μM solution of nitrite, prepare 5 vials of diluted nitrite solution across a range of micromolar concentrations.
    **CRITICAL:** Ensure that the highest and lowest concentrations used straddle the expected value of nitrite produced by the sonoreactor.
  c. Perform the Griess test in triplicate on each of the diluted samples.
  d. Using linear regression, calculate the relationship between the absorbance of each sample at ʎ_max_ = 540 nm versus concentration of nitrite.
    ***Note:*** An example of the calibration curve for nitrite ion quantification can be found in the Supplemental Information (Figure S1) of the associated primary research manuscript.^1^ Example UV-vis spectra that can be used to construct such a calibration are shown in Figure 6.

### Quantification of nitrate ions in water

**Timing: 1 h**

This step describes the method of nitrate ion determination through direct UV-vis absorption. The limit of detection for the procedure described below is 1.0 μM.

3. Measure the nitrate ion concentration in aqueous samples.
  a. Add 5 mL of the sample to be tested to a 10 mL vial.
  b. Add 0.1 mL of 1 M HCl to the vial and agitate for 1 minute.
  c. Allow the mixture to stand for 5 min.
  d. Collect a UV-vis spectrum of the sample between 300 nm and 200 nm, noting the absorbance at ʎ = 220 nm.

***Note:*** The direct UV-vis absorption method can be affected by the presence of dissolved organic matter. This should not be an issue when working with ultrapure water.

4. Prepare a calibration curve with solutions of known nitrate concentration, following the same steps as described for nitrite ions.

**Figure 7:**
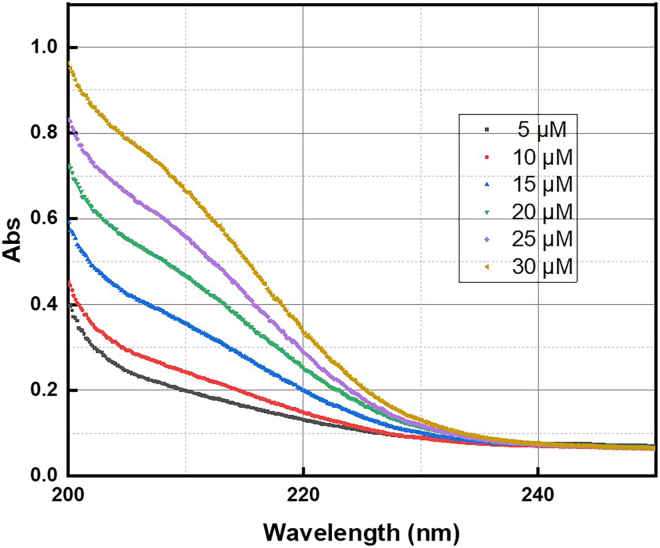
Example UV-vis spectra from the direct-UV calibration process for nitrate quantification The absorption at ʎ = 220 nm should be used for the calibration curve.

***Note:*** An example of the calibration curve for nitrate ion quantification can be found in the Supplemental Information (Figure S2) of the associated primary research manuscript.^1^ Example UV-vis spectra that can be used to construct such a calibration are shown in Figure 7.

### Quantification of *in*-*situ*-generated hydroxyl radicals

**Timing: 30 min**

These steps detail the general method for measuring the production of hydroxyl radicals in the sonoreactor through potassium iodide dosimetry. The concentration of triiodide is proportional to the extent of hydroxyl radical formation in the sonoreactor, and the method serves as a proxy for quantification.

5. Sonicate potassium iodide solutions to generate triiodide ions *in situ*.
  a. Fill the sonoreactor with 0.1 M potassium iodide solution.
  b. Perform the sonication experiment with the requisite conditions of power and pulse width, as previously performed with ultrapure water.
6. Collect a UV-vis spectrum of the sample from 400 – 300 nm, and record the absorbance (λ_max_) at 350 nm.
7. Using linear regression modelling of the absorbances of stock triiodide solutions, convert the absorbance at λ_max_ of the sample to moles of triiodide.

**Figure 8:**
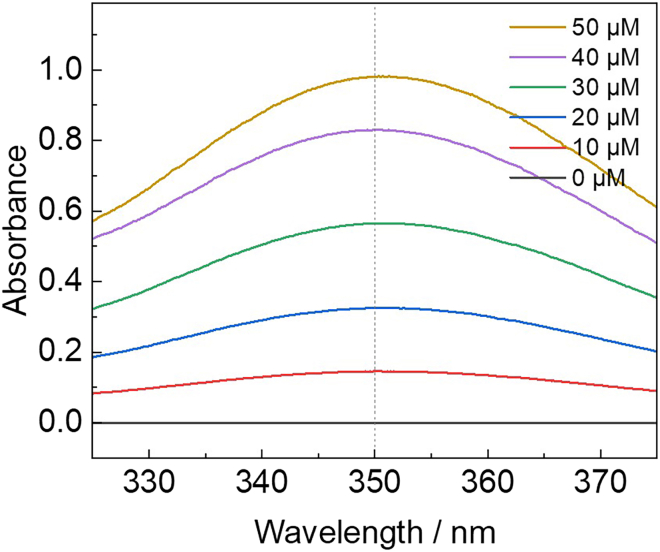
Example UV-vis spectra from solutions of triiodide, valid for both iodide dosimetry and H_2_O_2_ quantification The absorption at ʎ_max_ = 350 nm should be used for the calibration curve.

***Note:*** An example of the calibration curve for triiodide quantification can be found in the Supplemental Information (Figure S4) of the associated primary research manuscript.^1^ Example UV-vis spectra that can be used to construct such a calibration are shown in Figure 8.

### Quantification of *in*-*situ*-generated hydrogen peroxide

**Timing: 30 min**

These steps detail the general method for measuring the production of hydroxyl radicals in the sonoreactor through molybdate-catalyzed potassium iodide dosimetry.

8. Procedure for the molybdate-catalyzed dosimetry reaction.
  a. Into a UV-vis compatible 10 mm pathlength cuvette, add 3 mL of 0.1 M potassium iodide, 600 μL of the sonicated water sample, and 60 μL of 0.01 M ammonium molybdate solution.
  b. Leave the mixture to stand for five minutes.
9. Collect a UV-vis spectrum of sample from 400 – 300 nm and record the absorbance (λ_max_) at 350 nm.
10. Using linear regression modelling of the absorbances from the reaction performed on stock hydrogen peroxide solutions, convert the absorbance at λ_max_ of the sample to moles of hydrogen peroxide.

***Note:*** An example of the calibration curve for hydroxide quantification can be found in the Supplemental Information (Figure S3) of the associated primary research manuscript.^1^ The UV-vis spectra for these experiments will be similar in profile to those from the triiodide calibration.

### Optimization of the focused ultrasound sonoreactor

**Timing: 40 h**

This step involves the irradiation of aerated deionized water with high intensity focused ultrasound to form nitrate and nitrite anions. Different pulse durations and intervals are employed to find the optimum operating parameters.

11. Treat water samples with the sonoreactor, varying the parameters to find the optimum operating conditions.
  a. Ensure the sonoptic tank is filled with degassed water.
  b. Add 6 mL of ultrapure water to the sonoreactor.
  c. Flow air through the sonoreactor to ensure the water sample is saturated with air.
    ***Note:*** A flow rate of 19 liters per min for 10 min prior to sonication is sufficient.
  d. Program the reactor to sonicate the sample under the desired conditions of pulse width and pulse duration.
  e. After sonication, remove the sample from the sonoreactor and retain for nitrate and nitrite quantification (as described in Quantification Analysis)
  f. Perform the experiment in triplicate for each combination of pulse duration and pulse interval (e.g., pulse duration of 2, 4, 5, 7 ms, pulse interval of 20, 40, 80, 120, 160 ms).
    ***Note:*** Ensure that the energy supplied to the reactor across each set of operating parameters is constant by matching the number of bursts to the pulse duration. For example, a pulse duration of 2 ms with 10,000 bursts provides the same energy to the solution under irradiation as a pulse duration of 5 ms over 4,000 bursts.

### Processing acoustic data using MATLAB

**Timing: 10 min**

This step details the processing of acoustic data obtained using the passive cavitation detector using MATLAB in-built functions.

12. Process the acoustic data from the swPCD sensor with MATLAB.
  a. With MATLAB in-built functions, apply a band-pass filter (400 kHz – 4 MHz). This isolates the frequency range corresponding to the sensor’s sensitive bandwidth.
  b. Apply a high-pass filter with a 50 kHz cutoff to eliminate interference from cables.
  c. Apply a second high-pass filter with a 200 kHz cutoff to remove the fundamental driving frequency of the ultrasonic transducer.
  d. Use a bandpass filter to attenuate the harmonics of the driving frequency (i.e., integer multiples of the fundamental driving frequency).
  e. Obtain the root mean square value of the filtered signal to represent the time-averaged acoustic strength of acoustic cavitation within the reactor. This can be achieved with a command in the MATLAB environment as: rms(signal).

% swPCD post analysis

clear all; clc;

% N: number of burst (from Luk's code)

% PD1: Pulse duration taken for cavitation analysis [unit: sec]

% PD2: Pulse duration taken to find start of each pulse [unit: sec]

% fname = 'luk_006'; %loading file data saved as luk_006 in wfm format

%% load data

 [y, t, info, ind_over, ind_under, frames] = wfm2read_js(fname);

 dt = t(2,1)-t(1,1);

 fs = 1/dt;

 y = y ∗ 1000; % coverting to mV

%% processing (filtering to obtain shockwaves content.m)

 Fcut1 = 40e3;% Lower cutoff frequency

 Fcut2 = 4000e3;% Upper cutoff frequency; Fcut1 and Fcut2 are chosen based on swPCD characteristics

 N = number of burst

 s=cell(N,1); %Creating space

 sm=cell(N,1);%Creating space

 slpf1=cell(N,1);%Creating space

 slpf1y=cell(N,1);%Creating space

 slpf1yf=cell(N,1);%Creating space

 sig1=cell(N,1);%Creating space

 yrms=0;

 FS = fs;

 h_lpf = fir1(2ˆ9, 5e6/(fs/2));

 for i = 1:N

  s{i} = y(:,i);

 sm{i}=s{i}-mean(s{i}); % Removing the DC component

 slpf1{i} = convn(sm{i}, h_lpf', 'same');

  h_hpf = fir1(2ˆ9, 50/(fs/2), 'high');

  slpf1y{i} = convn(slpf1{i}, h_hpf', 'same');

  h_hpf = fir1(2ˆ9, 200000/(fs/2), 'high');% High pass at 200kHZ, source frequency

    slpf1yf{i} = convn(slpf1y{i}, h_hpf', 'same'); %Convolution of the filter with signal

  h_bpf = fir1(2ˆ9, [Fcut1 Fcut2]/(FS/2), 'bandpass');% Bandpass filter within active region of the swPCD

  sig1{i} = convn(slpf1yf{i}, h_bpf', 'same');

  yrms_luk(i) = rms(sig1{i}); % Computing the rms of all the burst

 end

### Estimating the acoustic power of the prototype sonoreactor

**Timing: 1 h**

This step involves the sonication of a sample of water in the bespoke prototype sonoreactor to measure the acoustic power density. This is an important parameter for comparing sonochemical setups, and is obtained by using the calorimetry method.

13. Track the temperature increase of a sample of water in the sonoreactor and calculate the acoustic power density.
  a. Add 20 mL of ultrapure water to the sonoreactor.
  b. Place a thermocouple at a fixed distance from the surface of the water (e.g., 5 mm)
  c. Sonicate the sample by energizing the reactor for a short period of time (e.g., 150 s), while plotting a graph of temperature versus time.
  d. Repeat this process a total of three times.
  e. Obtain the rate of change of temperature per unit time (e.g., with linear regression or by differentiating with respect to time).
  f. Calculate the acoustic power density *P*_*ac*_ with the following formula:

$$P_{ac}=\frac{dT}{dt}C_{p}M$$

Where *dT*/*dt* is the rate of temperature change in K/s, *C*_*P*_ is the specific heat capacity of water under the experimental conditions (i.e., 4.184 kJ/kg/K at 20°C), and *M* is the mass of water used.

**CRITICAL:** Ensure the reactor has completely cooled in between repeat experiments to ensure accuracy.

### Nitrogen fixation with the prototype sonoreactor

**Timing: 1 h**

This step utilizes the optimum operating parameters of pulse duration and interval (as determined with the optimization setup) and applies them to the prototype sonoreactor for larger bench-scale nitrogen fixation.

14. Sonicate samples of water in the prototype sonoreactor while employing the experimentally determined optimum pulse width settings.
  a. Add 20 mL of ultrapure water to the sonoreactor.
  b. Connect the cooling jacket of the sonoreactor to a temperature controlled water source (e.g., recirculating chiller or reservoir).
  c. Aerate the sample with compressed air for 10 mins at 19 L/min to ensure the water is saturated with air.
  d. Program the piezo driver to activate the transducer with the optimum pulse width parameters found with the focused ultrasound reactor.
  e. Sonicate the sample for the desired length of time, and retain for nitrate and nitrite ion quantification.

## Expected outcomes

The expected yield of nitrite and nitrate should be in the region of 20 and 40 μM respectively using the bespoke sonoreactor energized using an input protocol (PI = 80 ms; PD = 4 ms, and number of Bursts = 120,000) equivalent to 8 min active sonication time. Similarly, the expected yield from the focused transducer for nitrite and nitrate should be in the region of 1.3 and 11 μM respectively for 1 minute of active sonication time using the optimum input protocol (PI = 80 ms; PD = 4 ms, and # Bursts = 15,000).

## Limitations

Optimizations of the sonoreactor in terms of pulse width modulation can result in marked improvements in nitrate and nitrite yield. However, ultrasonic nitrogen fixation is limited by the efficiency of the transducer. With respect to joules required per mole of NO_x_ formed, the efficiency of this device (4.5 × 10^5^ kJ mol^−1^) is many orders of magnitude below the efficiency of established industrial processes such as the Haber-Bosch (485 kJ mol^−1^) and Birkeland-Eyde (3.6 × 10^3^ kJ mol^−1^) processes. Whatever the type of transducer, impedance matching is very important for maximum power transfer to the load. In all cases, the water sample should be ultra-pure and pre-sparged for at least 10 min before energizing the transducer.

It should also be noted that this protocol only details the formation and detection of nitrate and nitrite. Other nitrogenous species (e.g., NH_x_, organic N) may form in the presence of hydrogen or dissolved organics; however, this is unlikely when ultrapure water is used. Ion chromatography may be used for further validation and to broaden the scope of nitrogen fixation product analysis. For scalability of the process, the optimum sample volume (i.e., water to be put in the reactor) is very important for the efficiency of the sonoreactor. The larger the reacting volume, the farther the component height of the liquid volume from the ultrasound source and hence, the higher the attenuation. This, however, is not a linear case as other phenomena like sound interference could play a role.

## Troubleshooting

### Problem 1

Locating the ultrasonic transducer focus.

Running the focused ultrasound reactor with the sonoreactor outside of the focus region results in loss of efficiency. This may give lower than expected yields of nitrite and nitrate. Refer to the section detailing the setup of the focused ultrasound reactor.

### Potential solution

Map the sonoptic tank with a hydrophone to locate the focus point of the transducer.

• Energize the transducer at a very low pressure.
• Use a hydrophone to scan the acoustic field.
• Map the sonoptic tank in terms of acoustic intensity and note the maximum.
• Place your sonoreactor at the identified position for subsequent experiments.

### Problem 2

Unwanted cavitation in the sonoptic tank.

Sometimes cavitation forms outside the sonoreactor tube (i.e., within the water of the sonoptic chamber). This happens when the surrounding water is not well degassed, with dissolved gases acting as nucleation points for cavitation. This should not be encouraged as it can reduce the cavitation occurring in the sonoreactor. It could also cause erosion or cracking of the glass sonoreactor tube. Refer to the section detailing the preparation of the focused ultrasound reactor.

### Potential solution

Ensure that the degassed water that fills the sonoptic tank has been sufficiently degassed. A total dissolved gas sensor can be used to measure the extent of degassing. Make changes to the degassing method to ensure that no cavitation occurs outside the sonoreactor.

• Increase the length of time that the boiled water resides in the media bottle before use (e.g., 48 h instead of 24 h)
• Prepare the quantity of degassed water needed across fewer, larger media bottles (e.g., one 5 L bottle instead of five 1 L bottles) to improve the degassing process.
• Gently pour the degassed water into the sonoptic tank and avoid pouring from a distance.

### Problem 3

Lower power delivered to the bespoke sonoreactor.

The benchtop sonoreactor prototype is powered by a small transducer and may not perform as expected without the proper electrical input. Refer to the section detailing the preparation of the bespoke bench-scale sonoreactor.

### Potential solution

Carry out an impedance analysis for the sonoreactor to obtain the optimum parameters of impedance, frequency, and phase. This is similar to the process employed for the wiring and setup of the focused ultrasound transducer. Energize the device using the values found with this analysis. Ensure also that the transducer is being cooled sufficiently by the jacketed enclosure.

### Problem 4

Inconsistency or absence of detectable nitrite and nitrate in sonicated samples.

Certain pulse width settings will give very low yields of fixed nitrogen products. However, it is important to ensure consistency and repeatability across replicates (even though cavitation is a relatively stochastic phenomenon). Large errors in the yields of nitrate and nitrite from ultrasonic irradiation may suggest problems with the coupling of the ultrasound to the liquid in the sonoreactor. Refer to the section detailing the optimization of the focused ultrasound reactor.

### Potential solution

Check that the experimental conditions, particularly the aeration and positioning of the sonoreactor, are identical between replications of the sonication experiments. Test for the presence of hydrogen peroxide and triiodide (with iodometric dosimetry) in sonicated samples; high concentrations of hydrogen peroxide suggests that cavitation is occurring without nitrogen fixation. Check that the sample is sufficiently aerated before commencing sonication.

### Problem 5

Electrical shock/Device malfunctioning.

If the connecting wires are not well insulated or the device not properly earthed, it is possible for the operator to experience electric shock or for the device not to work effectively.

### Potential solution

Ensure that a well-trained expert helps with the electrical connections and ensure the wires are well insulated, and that the device is properly earthed.

#### Precautions

The following precautions should be taken.

• Wear complete personal protective equipment (PPE) before running any chemical experiment.
• Use ear defenders while operating the sonoreactor.
• Ensure you dispose of your chemical waste properly.

## Resource availability

### Lead contact

Further information and requests for resources and reagents should be directed to and will be fulfilled by the lead contact, Mark D. Symes (mark.symes@glasgow.ac.uk).

### Technical contact

Technical questions on executing this protocol should be directed to and will be answered by the technical contact, Lukman A. Yusuf (lukman.yusuf@glasgow.ac.uk).

### Materials availability

This study did not generate new unique data.

### Data and code availability

The data underpinning this study have been deposited in the University of Glasgow’s Enlighten database: https://doi.org/10.5525/gla.researchdata.1925.

## Acknowledgments

This work was supported by the 10.13039/501100000266EPSRC (EP/W037564/1). M.D.S. thanks the 10.13039/501100000288Royal Society for a University Research Fellowship (URF\R\211007).

## Author contributions

L.A.Y. and S.F. performed the experiments and analyzed the results. P.P. and M.D.S. conceived the idea and supervised the work. All authors contributed to the writing of the paper.

## Declaration of interests

The authors have filed a patent related to the original research manuscript.^1^
